# Co‐causation of reduced newborn size by maternal undernutrition, infections, and inflammation

**DOI:** 10.1111/mcn.12585

**Published:** 2018-01-08

**Authors:** Per Ashorn, Lotta Hallamaa, Lindsay H. Allen, Ulla Ashorn, Upeksha Chandrasiri, Megan Deitchler, Ronan Doyle, Ulla Harjunmaa, Josh M. Jorgensen, Steve Kamiza, Nigel Klein, Kenneth Maleta, Minyanga Nkhoma, Brietta M. Oaks, Basho Poelman, Stephen J. Rogerson, Christine P. Stewart, Mamane Zeilani, Kathryn G. Dewey

**Affiliations:** ^1^ Centre for Child Health Research, Faculty of Medicine and Life Sciences, University of Tampere and Tampere University Hospital Tampere Finland; ^2^ Department of Paediatrics Tampere University Hospital Tampere Finland; ^3^ USDA Agricultural Research Service Western Human Nutrition Research Center Davis California USA; ^4^ Program in International and Community Nutrition, Department of Nutrition University of California, Davis Davis California USA; ^5^ Department of Medicine at Peter Doherty Institute University of Melbourne Parkville Victoria Australia; ^6^ Food and Nutrition Technical Assistance III Project Washington DC District of Columbia USA; ^7^ Institute of Child Health University College London London UK; ^8^ Faculty of Public Health and Family Medicine, College of Medicine University of Malawi Blantyre Malawi; ^9^ External Research and Nutrition Nutriset S.A.S Malaunay France

**Keywords:** LAZ, low‐income countries, Malawi, newborn size, pathways, WAZ

## Abstract

More than 20 million babies are born with low birthweight annually. Small newborns have an increased risk for mortality, growth failure, and other adverse outcomes. Numerous antenatal risk factors for small newborn size have been identified, but individual interventions addressing them have not markedly improved the health outcomes of interest. We tested a hypothesis that in low‐income settings, newborn size is influenced jointly by multiple maternal exposures and characterized pathways associating these exposures with newborn size. This was a prospective cohort study of pregnant women and their offspring nested in an intervention trial in rural Malawi. We collected information on maternal and placental characteristics and used regression analyses, structural equation modelling, and random forest models to build pathway maps for direct and indirect associations between these characteristics and newborn weight‐for‐age Z‐score and length‐for‐age Z‐score. We used multiple imputation to infer values for any missing data. Among 1,179 pregnant women and their babies, newborn weight‐for‐age Z‐score was directly predicted by maternal primiparity, body mass index, and plasma alpha‐1‐acid glycoprotein concentration before 20 weeks of gestation, gestational weight gain, duration of pregnancy, placental weight, and newborn length‐for‐age Z‐score (*p* < .05). The latter 5 variables were interconnected and were predicted by several more distal determinants. In low‐income conditions like rural Malawi, maternal infections, inflammation, nutrition, and certain constitutional factors jointly influence newborn size. Because of this complex network, comprehensive interventions that concurrently address multiple adverse exposures are more likely to increase mean newborn size than focused interventions targeting only maternal nutrition or specific infections.

**Key messages**

- In low‐income conditions, maternal infections, inflammation, nutrition, and other factors jointly influence newborn size. Because of this complex network, interventions that address multiple adverse exposures concurrently are more likely to increase mean newborn size than the traditional narrowly focused interventions.
- Infections play a prominent role in predicting newborn length‐for‐age Z‐score and hence indirectly also newborn weight‐for‐age Z‐score.
- Holistic prevention and management interventions are needed to limit the adverse impact of maternal infections on the duration of pregnancy and foetal growth in low‐income settings.

## 1 INTRODUCTION

Of the approximately 135 million infants born each year, 16% (22 million) are born small, as indicated by a low birthweight (<2.5 kg; UNICEF, 2014). These babies have an increased risk of mortality, malnutrition, growth failure, and developmental delay in childhood and adverse health consequences in adult life (Christian et al., 2013; Katz et al., 2013; Lawn, Cousens, Zupan, & Lancet Neonatal Survival Steering Team, 2005). Therefore, prevention of low birthweight and small birth size is considered a public health priority, especially in the sub‐Saharan African and South Asian low‐income countries where their incidence is highest (UNICEF, 2014).

A small birth size can result either from a preterm birth or a restricted foetal growth velocity or a combination of these two. Both conditions are common in low‐income settings (Lee et al., 2013) and associated with such risk factors as maternal undernutrition, inflammation, and stress and with infections including malaria, HIV, and reproductive tract infections (Brodsky & Christou, 2004). Typical interventions to improve birth size have therefore included maternal dietary supplementation and infection treatment in pregnancy. Although some positive results have been reported from such interventions, the effect sizes have been modest, and many studies have reported no impact at all (Bhutta et al., 2013). One possible explanation for this limited success is narrowness of scope, that is, that research trials have addressed only one, or at maximum a few, of the putative risk factors. This approach assumes a single dominant aetiology for small birth size, but a co‐causation model is arguably more plausible, especially in low‐income contexts where infections, undernutrition, and other adverse exposures are often common among pregnant women. So far, however, there have been few studies that have concurrently analyzed multiple maternal risk factors and their joint contribution to birth size.

To characterize the determinants of birth size in a low‐income context, we carried out a large prospective study in rural Malawi, in which we collected information on maternal, foetal, placental, and newborn health at several points of pregnancy and thereafter. Using regression analysis, structural equation modelling (SEM), and random forest techniques, we built a concept map to characterize and illustrate the network of pathways connecting maternal constitutional variables, infection, inflammation, nutrition, stress, and the duration of pregnancy with newborn size. Because of its public health implications, we used newborn weight as the main outcome but also included newborn length in our models, to separate the contributions of foetal linear growth and fat and lean tissue deposition to the outcome of interest.

## 2 METHODS

### 2.1 Study design and general context

This was a prospective cohort study, nested within a randomized, outcome assessor‐blinded intervention trial in rural Malawi, carried out between February 2011 and April 2015 in an area with frequent maternal infections and a high prevalence of preterm birth and low birthweight (Ashorn, Alho, Ashorn, Cheung, Dewey, Gondwe, et al., 2015a; Luntamo et al., 2010). The original trial was designed to study the impact of small‐quantity lipid‐based nutrient supplements on maternal and child outcomes, when provided to women in pregnancy (enrolment <20 gestation weeks) and during the first 6 months of lactation and their newly born children from 6 to 18 months of age. In the embedded cohort study described in this article, we characterized the direct and indirect associations between selected maternal characteristics and newborn size, focusing on pathways that lead to reduced weight or length in the newborn.

According to the main results of this trial, named iLiNS‐DYAD‐M, provision of small‐quantity lipid‐based nutrient supplements to women in pregnancy and the first 6 months of lactation and to their children from 6 to 18 months of age was not associated with improved birth outcomes or child length or weight at 18 months of age (Ashorn, Alho, Ashorn, Cheung, Dewey, Gondwe, et al., 2015a; Ashorn, Alho, Ashorn, Cheung, Dewey, Harjunmaa, et al., 2015b).

The trial was performed according to Good Clinical Practice guidelines and the ethical standards of the Helsinki Declaration. The protocol was approved by the College of Medicine Research and Ethics Committee, University of Malawi and the Ethics Committee of Pirkanmaa Hospital District, Finland, and it was registered at the http://clinicaltrials.gov database (registration number NCT01239693). Only participants who signed or thumb‐printed an informed consent form were enrolled in the study.

For the currently described cohort study, we included all newborns who participated in the main trial, except twins and infants who had no postnatal anthropometric measurements.

### 2.2 Variables used for the models

**Figure 1:**
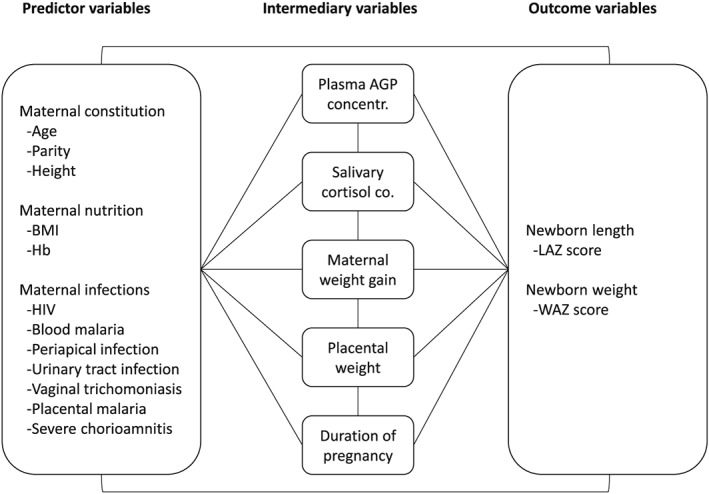
Conceptual model of pathways contributing to newborn size. Conceptual model of how maternal characteristics or maternal direct exposures during follow‐up (defined as predictor variables) could contribute to newborn size (outcome variables weight‐for‐age Z‐score [WAZ] and length‐for‐age Z‐score [LAZ]) either directly or through intermediary variables that follow from a direct exposure. Hb = haemoglobin; AGP = α‐1‐acid glycoprotein; BMI = body mass index

We based our pathway maps on a conceptual model, according to which maternal exposures (defined as predictor variables) could contribute to newborn size either directly or through intermediary variables that could also have an effect on each other (Figure 1). Thus, the predictor category included variables that described maternal characteristics in early pregnancy or the woman's direct exposures during the follow‐up (e.g., maternal nutrition or infection), whereas the intermediary variables were those that would follow from a primary exposure. The predictor and intermediate variables were selected either based on earlier literature (Brodsky & Christou, 2004) or because our own initial analyses had documented an association between them and newborn size (Harjunmaa et al., 2015; Stewart et al., 2015).

The variables in the predictor category covered maternal constitution and obstetric history (maternal age and height at enrolment and primiparity), maternal nutritional status (body mass index [BMI] and blood haemoglobin concentration), and maternal infections (HIV, peripheral blood malaria parasitemia at enrolment, and dental periapical infections, urinary tract infections, vaginal trichomoniasis, placental malaria, and severe chorioamnionitis at delivery or soon thereafter). Because newborn weight and length were measured soon after birth but not at birth (mean age 13 days), the delivery method (which may affect length immediately after birth) would be unlikely to affect them. Similarly, there would be little variation in feeding types at that point. Hence, we did not include the delivery mode or infant feeding variables in our models.

As intermediary variables, we selected plasma alpha‐1‐acid glycoprotein (AGP) concentration at enrolment as a sign of inflammation, salivary cortisol concentration at 28 gestation weeks as a sign of maternal stress, maternal weight gain during pregnancy, placental weight, and the duration of pregnancy (established using obstetric ultrasounds before 20 gestation weeks). For the size outcomes, we studied newborn weight‐for‐age Z‐score (WAZ) and length‐for‐age Z‐score (LAZ), measured at the latest by 6 weeks after birth. We were interested in weight because it predicts childhood wasting and mortality (Katz et al., 2013), and length both because it is a determinant of newborn weight and because it predicts childhood stunting, the reduction of which is a recent World Health Organization target (de Onis et al., 2013; Espo et al., 2002). To describe and visualize the predictors of newborn length (linear growth) and “robustness” (ponderal growth) and their contribution to newborn weight separately, we included LAZ in the models and concept maps for newborn WAZ. To establish and document the independent contribution of the duration of pregnancy on newborn size, we entered the duration as one variable and expressed the anthropometric indexes unadjusted to the duration of pregnancy—that is, we used the World Health Organization growth standard that adjusts for postnatal age but not for the duration of pregnancy, rather than the newer Intergrowth‐21st standard that adjusts birth size for pregnancy duration but not for postnatal age.

We assessed newborn anthropometrics at a clinic visit 1–6 weeks after delivery, rather than immediately at birth. This choice was motivated by the difficulty of accurately measuring length soon after birth and because a number of participants delivered at home, where length measurement would have been logistically difficult. To adjust the newborn size data for age at measurement (and for newborn sex), we calculated anthropometric indices (WAZ and LAZ) and used them in our models, instead of raw weight and length values (WHO Multicentre Growth Reference Study Group, 2006).

Further details of the data collection are provided in Data S1.

### 2.3 Statistical analyses

We started the analysis by establishing the bivariate associations (*p* < .05) between the predictor and intermediary variables that we anticipated to be associated with newborn WAZ and LAZ. We then built regression models for newborn WAZ and LAZ, adding multiple variables into the models one‐by‐one, with the perceived most distal predictor variables first and most proximal intermediary ones last. To identify potential indirect pathways, we built similar multivariable regression models also for each of the predictor and intermediary variables. Based on results from these regression models, we then created a graphic illustration for direct and indirect pathways contributing to newborn WAZ and LAZ. Finally, we made comparisons on how the various categories of predictor variables were associated with newborn WAZ or LAZ.

We imputed missing data for predictor variables and intermediary variables as well as outcome variables that were measured after 6 weeks from delivery. We used multiple imputed data (50 imputations) based on chained equation methods, for all ordinary least‐squares regression and logistic regression analyses. Among the included participants for regression analyses, the number of originally missing values that were substituted with values obtained by multiple imputation ranged from 0 to 480 (0.0% to 34.8%) per variable (Supplementary Table 1).

We estimated regression coefficients for continuous variables and odds ratios for dichotomous variables when estimating associations between variables in bivariate and multivariable models. We did not use normalization procedures for any variables because parametric analysis of means is robust and valid in case of a large sample size, regardless of the shape of outcome distribution as per the central limit theorem (Cheung, 2014). All models including salivary cortisol concentration were adjusted for time since waking up and time since last meal or drink. The intervention provided during pregnancy was not included as a covariate in the models because it was not statistically significant, and adding it in the models did not change the results.

We estimated coefficients for pathway models using SEM that allowed us to include variables both as endogenous (outcome) and exogenous (predictor) in the same model. The arrows pointing to the most central variable (LAZ and WAZ) describe the change in the outcome (Z‐scores for anthropometric indices). Arrows pointing to any other continuous variable in the model describe the change in standardized values (mean 0 and standard deviation [*SD*] 1). Continuous exogenous variables were also standardized. The coefficients of dichotomous exogenous variables are interpreted as the change in the outcome variable when the value of dichotomous variable changes from 0 to 1.

We used the maximum likelihood method with missing values to estimate model parameters in the SEM. We allowed correlation between all exogenous variables and calculated the χ^2^ value for the model. We also estimated three other goodness of fit indices for each SEM; the root mean square error of approximation (RMSEA), the comparative fit index (CFI), and the Tucker and Lewis index (TLI). An RMSEA score of zero indicates a perfect fit, scores <0.05 are considered markers of a good fit, and scores ≥0.05 and <0.08 are considered to indicate an adequate fit. For CFI and TLI, values at or above 0.90 are typically considered to indicate a well‐fitting model. All three goodness of fit indices adjust for the relative complexity of the model (Hu & Bentler, 1999; Kline, 2011).

In SEM, we used only continuous endogenous variables that in regression analyses were associated (*p* < .05) with newborn size. We considered *p* values below .05 indicative of statistical significance also in SEM. Because we could not estimate parameters for pathways leading to the dichotomous variables (maternal malaria, HIV, and primiparity) with SEM, we established the direction of association from regression models and indicate the associations with dashed lines in the pathway graphs.

As a sensitivity analysis to SEM, we used random forest modelling and forest plots (R Version 3.2.1, number of trees to grow 1,000). We imputed missing values in predictor data using R software's command rfImpute in random forest and regressed WAZ and LAZ against all the tested predictor and intermediate variables and calculated raw variable importance for all predictor and intermediate variables.

All statistical analyses were done with either Stata Version 13.0 (StataCorp, College Station, TX, USA) or R Version 3.2.1.

## 3 RESULTS

Of the 1,391 women who were enrolled into the iLiNS‐DYAD‐M study, 1,379 (99.1%) provided data for analyses with predictor and intermediary variables and 1,179 (84.8%) provided data for the analyses also with child anthropometrics. The reasons for exclusion were twin pregnancy (12 subjects) and lack of any anthropometric information for the infant (because of loss of pregnancy, death, or drop‐out; 200 subjects). The included women were less often primiparous than the excluded ones (19.5% vs. 35.1%). For other baseline characteristics, there were some statistically significant but clinically nonsignificant differences between the included and excluded participants (Table 1). Of the newborns, 91 (7.8%) had either length or weight values estimated by multiple imputation, because the actual values were collected only after 6 weeks of age. There were no clinically significant differences in the mean baseline characteristics between participants with imputed values for anthropometrics or any of the intermediary variables and participants with no imputed data for these variables (data not shown).

**Table 1:** Baseline characteristics at study enrolment of iLiNS‐DYAD trial participants who were included in or excluded from the current analyses^a^

Characteristic	Included	Excluded	*p* b
Number of participants	1,179	212	
Maternal age (years)	25 (6)	24 (6)	.002
Maternal education (completed years)	4.0 (3.4)	4.3 (3.7)	.340
Severely food insecure households (%)	36.0	35.7	.931
Gestational age (week)	16.8 (2.1)	17.1 (2.1)	.084
Number of previous pregnancies	2.2 (1.7)	1.7 (1.8)	<.001
Primiparous women (%)	19.5	35.1	<.001
Height (cm)	156.1 (5.7)	155.8 (5.7)	.438
Weight (kg)	53.9 (7.9)	55.2 (8.5)	.027
MUAC (cm)	26.3 (2.6)	26.6 (2.9)	.119
BMI (kg/m^2^)	22.1 (2.8)	22.7 (2.9)	.005
Women with a BMI < 18.5 kg/m^2^ (%)	5.5	4.7	.667
Blood haemoglobin concentration (g/L)	112 (16)	109 (16)	.010
Anaemic women (Hb < 100 g/L; %)	19.8	25.6	.055
Cortisol (nmol/L)	5.7 (2.9)	5.7 (3.8)	.811
Mean (SD) AGP (g/L)	0.8 (0.3)	0.7 (0.2)	.030
Women with AGP > 1 g/L (%)	12.5	14.1	.550
Women with a positive HIV test (%)	13.4	16.1	.358
Women with positive malaria RDT (%)	22.9	25.0	.500

*Note*. MUAC = mid‐upper arm circumference; Hb = haemoglobin; AGP = α‐1‐acid glycoprotein; RDT = rapid diagnostic test; BMI = body mass index.

a Values are means ± *SD* or percentages, unless otherwise indicated.

b The *p* value obtained from analysis of variance (continuous variables) or chi‐square test (proportions).

The mean (*SD*) age of the infants at size measurement was 13 (6) days. The mean (*SD*) newborn weight and length were 3.4 (0.5) kg and 49.7 (2.3) cm, corresponding to a WAZ of −0.56 (1.03) and LAZ of −1.00 (1.11). Of the babies born, 52.3% were boys, 6.3% were born preterm (<37.0 gestation weeks), 9.0% had a low newborn WAZ, and 30.6% were considered small‐for‐gestational age (Alexander, Himes, Kaufman, Mor, & Kogan, 1996). The mean (*SD*) duration of pregnancy at delivery was 39.6 (1.8) weeks, and the mean (*SD*) maternal weight gain and placental weight were 290 (100) g/week and 516 (102) g, respectively. Thirteen per cent (12.5%) of participants had an elevated AGP concentration (>1 g/L) at enrolment. Twenty‐three per cent (23.4%) had at least one dental periapical infection, 10.3% had vaginal trichomoniasis, 2.3% had a urinary tract infection, 11.8% had histologically documented severe chorioamnionitis, and 38.9% had signs of placental malaria. Mean maternal cortisol concentration at enrolment was 5.7 nmol/L and at 28 gestation weeks 5.9 nmol/L.

### 3.1 Predictors of the intermediary outcome variables

In multivariable models that included all the tested predictor and intermediary variables, plasma AGP concentration was independently associated with maternal primiparity, HIV infection, weight gain during pregnancy, peripheral blood malaria parasitemia at enrolment, and severe chorioamnionitis (Table 2). Maternal weekly weight gain, placental weight, and the duration of pregnancy were predicted by various combinations of the test variables (Table 2). None of the selected variables predicted maternal salivary cortisol concentration at 28 gestation weeks. These multivariable models explained 5.5–15.4% of the variance in intermediary variables.

**Table 2:** Multivariable regression model coefficients for predictors of the intermediary outcome variables^a^

	Intermediary outcome variable				
Predictor/intermediary variable	Plasma AGP	Salivary cortisol 28 weeks	Weekly weight gain	Placental weight	Duration of pregnancy
Maternal age^b^ (year)	−0.022^a^	−0.055	0.048	−0.004	0.013
Maternal primiparity^b^	0.374***	0.016	−0.145	−0.122	−0.026
Maternal height^b^ (cm)	−0.016	0.007	0.106***	0.103**	0.014
Maternal BMI^b^ (kg/m^2^)	−0.028	−0.010	−0.141***	0.099**	−0.056*
Maternal Hb^b^ (g/L)	−0.036	−0.001	0.061*	−0.069*	0.104***
Maternal HIV infection^b^	0.431***	0.027	−0.235**	−0.205*	0.024
Maternal malaria infection^b^	0.476***	−0.063	−0.118^a^	0.085	−0.134*
Maternal periapical infections^c^	0.013	0.000	−0.148*	0.091	−0.216*
Maternal urinary tract infection^c^	0.072	0.293	−0.089	0.138	−0.440*
Maternal trichomoniasis^c^	0.172^a^	−0.042	−0.096	−0.058	−0.153
Placental malaria infection^c^	0.123^a^	0.037	−0.143*	0.068	0.060
Severe chorioamnionitis^c^	−0.260**	0.001	0.030	−0.017	−0.349*
Maternal plasma AGP concentration^b^ (g/L)	N/A	0.030	0.093**	−0.054	−0.019
Maternal salivary cortisol concentration^d^ (nmol/L)	0.027	N/A	−0.057^a^	−0.006	−0.001
Maternal weekly weight gain^e^ (g/week)	0.085**	−0.057^a^	N/A	0.078*	0.024
Placental weight^c^ (g)	−0.050	−0.006	0.079*	N/A	0.280***
Duration of pregnancy^e^ (week)	−0.019	−0.001	0.026	0.299***	N/A
Adjusted coefficient of determination^f^ (*R* ^2^; %)	15.4	5.5	7.4	12.4	15.1

*Note*. Hb = haemoglobin; AGP = α‐1‐acid glycoprotein; BMI = body mass index.

a Values are β coefficients unless otherwise specified.

b Measured at study enrolment.

c measured at delivery or soon thereafter.

d measured at 28 gestation weeks.

e measured throughout the pregnancy.

f proportion of the dependent variable explained by the independent variables in the model, adjusted for the number of independent variables in the model.

* .01 ≤ *p* < .05.

** .001 ≤ *p* < .010.

*** *p* < .001.

# .050 ≤ *p* < .100.

### 3.2 Multivariable regression models of newborn weight‐for‐age and length‐for‐age

A multivariable regression model, adjusting for all the predictor variables, indicated that newborn WAZ was positively associated with maternal height, BMI, and haemoglobin (Hb) at enrolment and negatively associated with maternal primiparity, HIV infection, malaria, periapical infections, and urinary tract infection (Table 3, Model 1). Addition of the potential intermediary outcomes in the model removed the association with Hb and HIV infection and attenuated other coefficients but not maternal BMI (Table 3, Model 2). Of the intermediary variables, all but maternal salivary cortisol concentration were associated with newborn WAZ. Further adjustment with newborn LAZ left maternal primiparity, BMI and plasma AGP, and maternal weekly weight gain, placental weight, duration of pregnancy, and newborn LAZ as direct, statistically significant predictors of newborn WAZ (Table 3, Model 3). The fully adjusted multivariable model explained 64.1% of the variance in WAZ.

**Table 3:** Direct predictors of newborn LAZ and WAZ; results from multivariable regression models^a^

Predictor/intermediary/outcome variable/*R* ^2^	Newborn WAZ‐score			Newborn LAZ‐score	
	Model 1	Model 2	Model 3	Model 4	Model 5
Maternal age^b^ (year)	0.031	0.007	0.006	0.028	0.002
Maternal primiparity^b^	−0.385***	−0.287***	−0.139*	−0.373***	−0.285**
Maternal height^b^ (cm)	0.217***	0.138***	0.024	0.288***	0.220***
Maternal BMI^b^ (kg/m^2^)	0.065*	0.075**	0.053*	0.030	0.043
Maternal Hb^b^ (g/L)	0.090**	0.024	0.024	0.068*	−0.001
Maternal HIV infection^b^	−0.193*	−0.039	0.046	−0.305**	−0.164#
Maternal malaria infection^b^	−0.225**	−0.125*	−0.072	−0.195**	−0.102
Maternal periapical infections^c^	−0.193*	−0.133*	−0.025	−0.271**	−0.207**
Maternal urinary tract infection^c^	−0.452*	−0.344*	−0.186	−0.405^a^	−0.305
Maternal trichomoniasis^c^	−0.167^a^	−0.08	−0.075	−0.081	−0.009
Placental malaria infection^c^	−0.091	−0.055	−0.010	−0.112	−0.085
Severe chorioamnionitis^c^	−0.096	−0.063	−0.020	−0.118	−0.083
Maternal plasma AGP concentration^b^ (g/L)		−0.107***	−0.056*		−0.099**
Maternal salivary cortisol concentration^d^ (nmol/L)		0.017	0.012		0.011
Maternal weekly weight gain^e^ (g/week)		0.090***	0.051**		0.075**
Placental weight^c^ (g)		0.303***	0.181***		0.236***
Duration of pregnancy^e^ (week)		0.680***	0.342***		0.652***
Newborn LAZ‐score			0.519***		
Adjusted coefficient of determination (*R* ^2^; %)^f^	13.5	43.5	64.1	13.6	34.3

*Note*. WAZ = weight‐for‐age Z‐score; LAZ = length‐for‐age Z‐score; Hb = haemoglobin; AGP = α‐1‐acid glycoprotein; BMI = body mass index.

a Values are β coefficients unless otherwise specified.

b Measured at study enrolment.

c Measured at delivery or soon thereafter.

d Measured at 28 gestation weeks.

e Measured throughout the pregnancy.

f Adjusted coefficient of determination = proportion of the dependent variable explained by the independent variables in the model, adjusted for the number of independent variables in the model.

* .01 ≤ *p* < .05.

** .001 ≤ *p* < .010.

*** *p* < .001.

# .050 ≤ *p* < .100.

Newborn LAZ was positively associated with maternal height and Hb concentration at enrolment and negatively associated with maternal primiparity, HIV infection, peripheral blood malaria parasitemia at enrolment, and dental periapical infections (Table 3, Model 4). Adjusting the analysis further with the potential intermediary variables removed the association with Hb and malaria and attenuated the other coefficients. Of the potential intermediary variables, maternal plasma AGP concentration, weekly weight gain, placental weight, and duration of pregnancy, but not maternal salivary cortisol concentration, were all directly associated with newborn LAZ (Table 3, Model 5). The model explained 34.3% of the variance in LAZ.

In the regression analyses, newborn LAZ and WAZ were thus both directly predicted by maternal primiparity and four of the predefined intermediary variables, that is, the duration of pregnancy, placental weight, maternal plasma AGP, and maternal weight gain during pregnancy. Newborn LAZ was additionally predicted by maternal height, dental periapical infections, and HIV infection (*p* = .058), whereas newborn WAZ was predicted by maternal BMI at enrolment and newborn LAZ but not directly by maternal infections.

### 3.3 Pathways of causality

**Figure 2:**
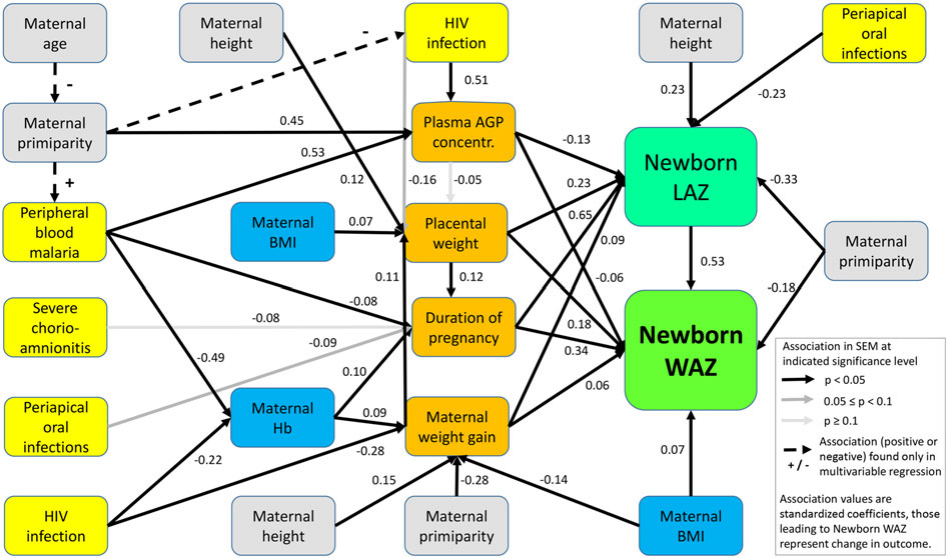
Pathway model for the determinants of newborn weight‐for‐age Z‐score (WAZ) and length‐for‐age Z‐score (LAZ). A structural equation model (structural equation modelling [SEM]), using variables that in regression analyses were associated with (*p* < .05) newborn size (green boxes) or defined intermediary variables (orange boxes). The predictor variables represent maternal nutrition (blue boxes), infections (yellow), or other constitutional variables (grey). The model χ^2^(36) = 117.92 (*p* < .001), *N* = 1,179 participants. Hb = haemoglobin; AGP = α‐1‐acid glycoprotein; BMI = body mass index

A pathway map, obtained from SEM and illustrating both the direct and indirect predictors of newborn size and coefficients between variables, is shown graphically in Figure 2. As with the regression models, newborn WAZ was directly predicted by newborn LAZ and maternal BMI at enrolment. Maternal primiparity and four of the predefined intermediary variables were directly associated both with WAZ and LAZ, but the coefficients were bigger for LAZ. Maternal height and dental periapical infections predicted newborn LAZ directly but WAZ only indirectly through newborn LAZ or maternal weight gain or placental weight. All the other test variables shown in the concept map were associated with newborn WAZ indirectly, sometimes through several pathways (Figure 2). The indicated model had a good fit (RMSEA = 0.044, CFI = 0.958, TLI = 0.918), and it explained 32.8% of the variance in newborn WAZ.

**Figure 3:**
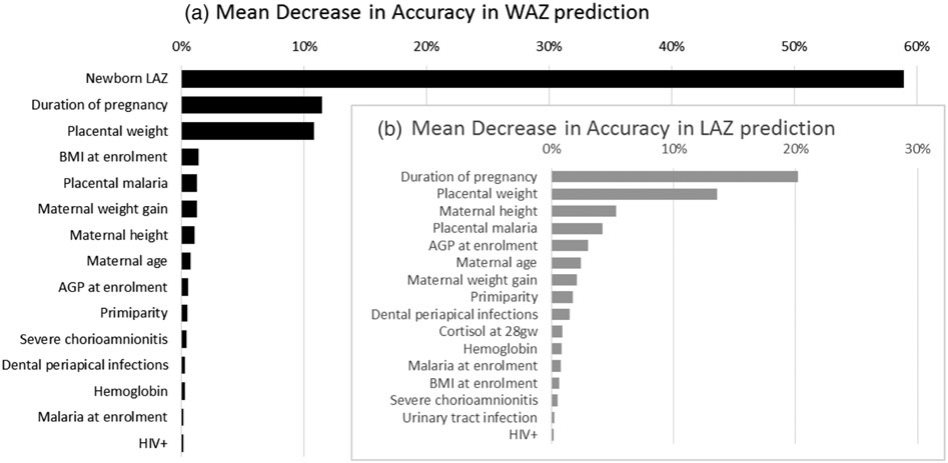
Random forest modelling of newborn weight‐for‐age Z‐score (WAZ) and length‐for‐age Z‐score (LAZ). In random forest modelling newborn LAZ, duration of pregnancy and placental weight are immediate predictors of newborn WAZ (Panel A—black bars), and Newborn LAZ was most strongly predicted by the duration of pregnancy and placental weight (Panel B—grey bars). AGP = α‐1‐acid glycoprotein; BMI = body mass index; gw = gestation weeks

Random forest models, including all the tested predictor and intermediate variables, confirmed the importance of newborn LAZ, duration of pregnancy, and placental weight as immediate predictors of newborn WAZ (Figure 3). When these variables were included in the model, each of the other variables accounted for less than 1.5% of the prediction accuracy for the newborn WAZ model. The duration of pregnancy and placental weight was the strongest predictors of newborn LAZ, but there were seven additional variables (including maternal height and placental malaria) that each accounted for at least 1.5% of the prediction accuracy for the newborn LAZ model (Figure 3).

## 4 DISCUSSION

The current analysis was done to characterize the determinants of newborn size in a rural Malawian setting, using newborn WAZ as the main indicator and newborn LAZ as a secondary size variable. In a sample of 1,179 pregnant women, newborn WAZ was directly predicted by maternal primiparity, BMI and plasma AGP concentration at enrolment, gestational weight gain, duration of pregnancy, placental weight, and newborn LAZ. The latter five variables were interconnected and predicted also by more distal determinants, so that newborn WAZ was ultimately associated with a wide range of variables reflecting maternal nutrition, infection, inflammation, and some constitutional factors including parity and height.

The methodological strengths of the study include a prospective study design, comprehensive data collection that included both clinical and laboratory variables, implementation in an area with high prevalence of preterm birth and low birthweight, and rigorous quality assurance in data collection. Internal validity could have been compromised by missing data, the delay in anthropometric measurements of some participants, and the choice of modelling techniques. External validity could have been further affected by the choice of variables in the pathway analyses and by the relative under‐representation of primiparous women in the sample. However, the excluded participants and those with missing data had on average similar baseline characteristics to participants who provided data, and we used multiple imputations to estimate the missing values in regression and SEM. We also included in our models variables that had been considered appropriate in earlier analyses (Bhutta et al., 2013; Brodsky & Christou, 2004; Goldenberg, Culhane, Iams, & Romero, 2008) and employed up‐to‐date statistical methods to examine relationships between several variables simultaneously (DiLalla, 2008). Finally, the results were robust to sensitivity analyses with random forest modelling. Therefore, although the data incompleteness certainly reduces the precision of our model estimates, we believe that the findings are valid and reliable and can be used to infer causal pathways associating the indicated exposures to newborn size in the population from which the sample was drawn.

A small newborn size can result from a preterm birth or a restricted foetal growth velocity or a combination of these two. Maternal nutrition, reproductive tract infections, chorioamnionitis, malaria, inflammation, and stress have been associated with preterm birth and hence small newborn size in low‐income settings in previous studies (Goldenberg et al., 2008). Factors associated with foetal growth restriction have typically included maternal anaemia, undernutrition, HIV and malaria infections, placental pathology, and foetal factors such as multiple gestation, genetic aberrations, or congenital viral infections (Brodsky & Christou, 2004; Hendrix & Berghella, 2008; Resnik, 2002). Our results corroborate these earlier findings and provide a model of how a multitude of factors jointly contribute to newborn size in one rural population in sub‐Saharan Africa.

One important finding from our study was the prominent role of infections in predicting newborn LAZ and hence indirectly also newborn WAZ. There are at least three possible mechanisms that might explain these associations. First, a local inflammatory response can weaken amniotic membranes and causes their premature rupture and preterm labour (Goldenberg et al., 2008). Second, inflammation typically activates prostaglandin synthesis, which may lead to increased contractibility of the uterus and softening and shortening of the cervix, also inducing preterm labour (Challis, Matthews, Gibb, & Lye, 2000). Third, infections can elicit a systemic inflammation that can restrict foetal growth at least through a reduction in placental vascularization or a diminished nutrient or oxygen transfer (Boeuf et al., 2013; Conroy et al., 2013).

In addition to its effects on placental vascularization and function, systemic inflammation has been shown to alter a hormonal pathway that drives the elongation of long bones in human fetuses and children. In a normal pregnancy, foetal growth is largely driven by peptide‐hormones called insulin‐like growth factors (IGFs) that are secreted by maternal tissues, the placenta, and the fetus (Woods, Camacho‐Hubner, Savage, & Clark, 1996). Both maternal and placental infection can be associated with downregulation of IGF expression in the placenta, low IGF concentrations in maternal, amniotic cord and newborn plasma, and higher concentrations of specific binding proteins that inactivate IGFs in foetal circulation (Dimasuay, Boeuf, Powell, & Jansson, 2016). These effects are at least partially mediated by pro‐inflammatory cytokines, such as IL‐1, IL‐6, and TNF‐alpha that interrupt signal transduction for IGF expression and can cause chondrocyte death in the growth plates of long bones (Hsiao & Patterson, 2011; Walters & Griffiths, 2009). The critical role of IGFs in foetal growth restriction has been confirmed in murine and rabbit models, where overexpression of the IGF‐1 gene in placental tissue has prevented experimentally induced intrauterine growth restriction in the fetus (Jones, Crombleholme, & Habli, 2013; Keswani et al., 2015).

The presented pathway model explained only one third of the variation in the study sample's newborn WAZ. This may be related to the lack of data on several exposures that have been shown to influence foetal growth or the duration of pregnancy, such as maternal periconceptional health, inflammation and micronutrient status, energy and nutrient intake during pregnancy, vascular disease, pre‐eclampsia and placental insufficiency, gestational diabetes, phospholipid‐immunity, bacterial vaginosis, genetics, and smoking or alcohol consumption during pregnancy (Goldenberg et al., 2008). Furthermore, we did not use repeated measurements, even for variables that are likely to undergo changes during pregnancy. Because of these limitations, the presented model cannot be considered a complete picture of newborn size determinants, even in the study sample. Nevertheless, it presents a template for how maternal infections, inflammation, undernutrition, and certain constitutional factors can jointly influence newborn weight in rural Malawi.

Because of the complex network of adverse exposures, it is not surprising that tightly focused antenatal interventions targeting only maternal nutrition or specific infections have at best had only modest impacts on foetal growth or duration of pregnancy in low‐income settings (Ashorn, Vanhala, Pakarinen, Ashorn, & De Costa, 2015; Bhutta et al., 2013). For a greater impact, it is likely that more comprehensive interventions will be needed, to ensure good nutritional status for the mother both before and during pregnancy, prevention and treatment of a wide range of maternal infections and possibly also the prevention of maternal stress. Promotion of good nutrition is already included in guidelines for antenatal care, but infection control in low‐income settings typically covers only malaria, HIV, and syphilis treatment. To further limit the impact of infection, the current study suggests that severe chorioamnionitis and maternal dental infections should also be targeted. There is, however, no reason to believe that this list of infections is exhaustive. Additional maternal infections that could be targeted during antenatal care could include for instance urinary tract infections, vaginal trichomoniasis, intestinal parasites, influenza, other viral infections, and environmental enteric dysfunction (Fell et al., 2017; Tellapragada et al., 2016). Further studies should be conducted to identify viral, parasitic, or bacterial infections that elicit systemic inflammation in pregnant women and contribute to adverse newborn outcomes in low‐income settings.

## CONFLICTS OF INTEREST

M. Z. works as a director of research for Nutriset S.A.S, a company that produces and sells lipid‐based nutrient supplements and which also prepared the LNS supplements purchased for the current trial. The other authors have no financial relationships relevant to this article to disclose. The findings and conclusions contained within the article are those of the authors and do not necessarily reflect positions or policies of the USAID, the United States Government, the Bill & Melinda Gates Foundation, or the other funders. One of the co‐authors (MD) is employed by FHI 360, which provided USAID funding for the study to UC Davis through the FANTA Project. The other funders had no role in the study design, data collection and analysis, decision to publish, or preparation of the manuscript. The corresponding author (PA) had full access to the data and final responsibility for the decision to submit for publication.

## CONTRIBUTIONS

PA, UA, MD, KM, KD, and MZ conceptualized and designed the study and the data collection. PA, UA, UH, KM, and MN supervised and coordinated collection of clinical data that were used in the study. LA, UC, RD, JJ, SK, NK, BO, SR, and CS carried out and supervised the conduct and interpretation of laboratory analyses that were used in the study. LH and BP conducted statistical analyses and created the tables and figures included in the manuscript. PA drafted the initial manuscript. All authors reviewed and revised the text and approved the final manuscript as submitted.

## Supporting information

Data S1: Supplementary Appendix
