# Supplementary material for: Co‐causation of reduced newborn size by maternal undernutrition, infections, and inflammation
Source: Matern Child Nutr. 2018 Jan 8;14(3):e12585. doi: 10.1111/mcn.12585 (PMC6055652; doi:10.1111/mcn.12585)
Supplement: Supplementary file 1 — Data S1: Supplementary Appendix [file MCN-14-e12585-s001.docx]

**Supplementary Material**

**Details of data collection**

We calculated maternal BMI from weight and height measurements conducted at the enrolment visit from all women who enrolled in the trial. Additionally, we measured weight at 32 and 36 gestational weeks to estimate weekly gestational weight gain from time of enrolment to 36 weeks gestation. We used mixed modeling to estimate the weekly weight gain (kg/week) for all participants with at least one weight measurement done between enrolment and 36 gestation weeks.

Clinic nurses collected the blood samples at enrolment and 36 gw. Laboratory technicians separated plasma by centrifugation of heparinized blood and stored it at -80^o^C. We later analyzed AGP concentration from those blood samples by immunoturbidimetry on the Cobas Integra 400 system autoanalyzer (F. Hoffmann-La Roche Ltd, Basel, Switzerland). Clinic nurses measured hemoglobin (Hb) concentration with disposable cuvettes (HemoCue, Ängelholm, Sweden), from whole blood collected from a finger prick at enrolment.

Research assistants collected saliva samples at enrolment, 28 gw and 36 gw between 8 am and 4 pm, with a mean collection time at approximately 11 am. Women were instructed not to consume any food or drink besides water for at least 30 minutes before providing the saliva sample. Time of saliva collection, time of waking, and time of last food or drink were recorded. Saliva samples were collected at clinic sites when women came to provide blood and urine samples and had anthropometric measurements taken. Saliva collection occurred before any other measurements or sample collection. Saliva was obtained by having the woman place an inert polymer cylindrical swab (10 mm x 30 mm, Salimetrics Oral Swab) under her tongue for approximately two minutes, while moving her tongue and jaw as if she were chewing to stimulate saliva. The swab was then placed in a tube with a cap and refrigerated or placed on ice packs. Swabs were brought to room temperature before centrifuging for 15 minutes at 3,000 RPM (1500 x g). Samples were frozen and stored at -20°C within 24 hours of collection. Thawed samples were later tested at the University of California, Davis using commercially available ELISA kits (Salimetrics, State College, PA).

Malaria was tested by rapid diagnostic testing (RDT) and by PCR. A finger prick sample was used for RDT at the study sites at enrolment, 32 gw and 36 gw. For PCR testing, whole blood was drawn by venipuncture at enrolment. Drops of blood were placed on filter paper to prepare dried blood spots (DBS) at the study clinic. The DBS samples were then shipped to the University of North Carolina where PCR analyses were conducted. The woman was determined to be malaria positive if either RDT of PCR indicated malaria at enrolment.

HIV testing and counseling was conducted according to national guidelines. HIV testing was offered to all women attending the antenatal clinic at the study sites. Pre-test HIV counseling was offered to all the women during the routine antenatal health talk. For the women who expressed interest in taking part in the study and did not opt out of the test, capillary blood was drawn by a finger prick and HIV tests were performed in the study rooms. Post-test counseling was offered to all women after conducting the test. Women who tested negative or had an indeterminate result were asked to return to the clinic for repeat testing after three months. Those who tested positive were referred to the antiretroviral clinic for treatment in accordance with Option B+ treatment guidelines for HIV positive pregnant women.

Research nurses or laboratory technicians weighed the placentas as soon as possible after delivery with dietary scales, with a reading increment of 1 g.

At one week after delivery the participants visited the study clinic for reproductive tract infection and urinary tract infection testing. A study nurse obtained a blind vaginal swab and immediately sent the sample to the study laboratory. The woman was also asked to provide a urine sample in a screw top bottle. The study nurse performed a urine dipstick analysis on the urine sample.

Two specifically trained dental therapists conducted a comprehensive clinical and questionnaire-based oral health assessment and took digital radiographs at the postnatal visit at one week after delivery or as soon as possible at Mangochi central site. The examiners’ measurement reliability was assessed and verified at the beginning and regularly during the study. An oral and maxillofacial radiologist and an experienced dentist jointly analyzed the radiographs using structured forms.

**Details of variables used in the models.**

For the size outcomes, we used newborn weight-for-age Z-score (WAZ) and length-for-age Z-score (LAZ), measured at the latest by 6 weeks after birth, as weight predicts childhood wasting and mortality, and length is both a determinant of newborn weight and it predicts childhood stunting. We treated the outcomes as continuous (e.g. mean WAZ) rather than dichotomous (e.g. WAZ<-2) variables, because a) it increased the power of the study, b) we were interested in birth size determinants over the entire range of value distribution (and not only a change from above to below a defined cut-off), and c) SEM models indicate effect size only for continuous outcomes.

Research assistants took triplicate measurements of length and weight with high-quality length boards (Harpenden Infantometer, Holtain Limited, Crosswell, Crymych, UK) and digital scales (SECA 381 baby scale, Seca GmbH & Co., Hamburg, Germany). For all the measurements, we used the mean of the first two readings if they did not differ by more than a pre-specified tolerance limit of 0.5 cm or 100 g. If the difference was above the limit, the third measurement was compared with the first and second measurements, and the pair of measurements that had the smallest difference was used to calculate the mean. If there were only one or two repeated measurements, the mean of those was used for the analyses.

We calculated the duration of pregnancy by adding the time interval between enrolment and delivery to the ultrasound-determined gestational age at enrolment. We estimated maternal mean weekly weight gain (g/week) during pregnancy by mixed modelling for all participants with at least one weight measurement done between enrolment and 36 gestation weeks. Mixed models can be used to estimate parameters for those with only one measurement without imputing the missing data because the method adjusts for the effect of missing values by using the correlation between repeated measurements (Cheung, 2014). Research assistants determined the participants’ blood hemoglobin concentration (Hb) with disposable cuvettes (HemoCue, Ängelholm, Sweden), collected and stored saliva and heparinized plasma at -80^o^C and measured placental weights immediately after delivery with digital scales. We later measured plasma AGP and salivary cortisol concentrations from stored biospecimens using immunoturbidimetry on the Cobas Integra 400 system autoanalyzer (F. Hoffmann-La Roche Ltd, Basel, Switzerland) and ELISA methods (expanded range high sensitivity salivary cortisol kit, Salimetrics, State College, PA), respectively. We considered AGP concentration above 1 g /L as high. Research assistants collected data on maternal age and parity by interview, maternal nutritional status by anthropometry and maternal infections by rapid point-of care tests (HIV, peripheral blood malaria, urinary tract infections), and microscopy of vaginal mucus (trichomoniasis). Further information on maternal infections was obtained through the review of histological slides (severe chorioamnionitis, placental malaria), DNA-amplification and detection from stored dried blood (peripheral blood malaria), or review of panoramic radiographs (dental periapical infections).

Values for some variables were collected several times during the pregnancy, but in our models we used them only from one time point. For the predictor variables that we assumed would affect the outcomes early in pregnancy or throughout it, we primarily used data collected at baseline. For logistical reasons, assessment of maternal dental periapical infections, urinary tract infection, vaginal trichomoniasis, placental malaria, and chorioamnionitis was performed only after delivery, but since these infections are typically chronic, we assumed that the postnatal data reflected the presence of the respective exposure during pregnancy. For the intermediary outcomes, we mostly used data collected towards the end of pregnancy, since we expected the values to change during follow-up. As intermediary outcomes, we thus used salivary cortisol concentration measured at 28 gestation weeks, maternal mean weekly weight gain throughout pregnancy, placental weight, and duration of pregnancy. An exception to this rule was plasma AGP concentration, which we used as a measure of maternal inflammation. For this variable, we used baseline and not 36-gestation week values, since late pregnancy is associated with an inflammatory process, often reflected in increased AGP values (Bollapragada et al., 2009; Taguchi, Nishi, Chuang, Maruyama, & Otagiri, 2013).

REFERENCES

References

Bollapragada, S., Youssef, R., Jordan, F., Greer, I., Norman, J., & Nelson, S. (2009). Term labor is associated with a core inflammatory response in human fetal membranes, myometrium, and cervix. *American Journal of Obstetrics and Gynecology, 200*(1), 104.e1-104.11.

Cheung, Y. B. (2014). *Statistical analysis of human growth and development*. FL.: CRC Press: Boca Raton.

Hu, L., & Bentler, P. M. (1999). Cutoff criteria for fit indexes in covariance structure analysis: Conventional criteria versus new alternatives. *Structural Equation Modeling: A Multidisciplinary Journal, 6*(1), 1-55.

Kline, R. (2011). *Principles and practice of structural equation modelling, 3rd ed* Guilford Press.

Taguchi, K., Nishi, K., Chuang, V. T. G., Maruyama, T., & Otagiri, M. (2013). Molecular aspects of human alpha-1 acid glycoprotein — structure and function, acute phase proteins, prof. sabina janciauskiene (ed.). *Intech,*

Supplementary table 1. Variables used in the analyses^a^

|  | |  | Variable type^b^ |  | Imputed variables | |  | Bivariate association with:^c^ | |
| --- | --- | --- | --- | --- | --- | --- | --- | --- | --- |
| Category and variable | |  |  |  | # | % |  | LAZ | WAZ |
| Maternal constitutional characteristics | | | | | | | | | |
|  | Age^d^ |  | C |  | 0 | 0.0 |  | 0.119*** | 0.148*** |
|  | Primiparity^d^ |  | D |  | 3 | 0.2 |  | -0.517*** | -0.569*** |
|  | Height^d^ |  | C |  | 5 | 0.4 |  | 0.306*** | 0.237*** |
| Maternal nutrition | | | | | | | | | |
|  | BMI^d^ |  | C |  | 9 | 0.7 |  | 0.061^a^ | 0.103** |
|  | Blood hemoglobin concentration^d^ |  | C |  | 2 | 0.1 |  | 0.138*** | 0.166*** |
|  | Weekly weight gain^g^ |  | C |  | 2 | 0.1 |  | 0.205*** | 0.211*** |
| Maternal infections | | | | | | | | | |
|  | HIV-infection^d^ |  | D |  | 57 | 4.1 |  | -0.272** | -0.150 |
|  | Peripheral blood malaria parasitaemia^d^ |  | D |  | 25 | 1.8 |  | -0.334*** | -0.396*** |
|  | Dental periapical infection^e^ |  | D |  | 353 | 25.6 |  | -0.208* | -0.115 |
|  | Urinary tract infection^e^ |  | D |  | 167 | 12.1 |  | -0.632* | -0.643** |
|  | Vaginal trichomoniasis^e^ |  | D |  | 165 | 12.0 |  | -0.263* | -0.308** |
| Maternal inflammation and stress | | | | | | | | | |
|  | Salivary cortisol concentration^f^ |  | C |  | 480 | 34.8 |  | -0.020 | -0.017 |
|  | Plasma AGP concentration^d^ |  | C |  | 8 | 0.6 |  | -0.180*** | -0.196*** |
| Placental weight, infection and inflammation | | | | | | | | | |
|  | Placental weight^e^ |  | C |  | 413 | 29.9 |  | 0.367*** | 0.428*** |
|  | Placental malaria infection^e^ |  | D |  | 371 | 26.9 |  | -0.309*** | -0.325*** |
|  | Severe chorioamnionitis^e^ |  | D |  | 389 | 28.2 |  | -0.156 | -0.121 |
| Duration of pregnancy and newborn size | | | | | | | | | |
|  | Duration of pregnancy^g^ |  | C |  | 84 | 6.1 |  | 0.799*** | 0.845*** |
|  | Newborn LAZ-score^e^ |  | C |  | 80 | 6.8 |  |  | 0.694*** |
|  | Newborn WAZ-score^e^ |  | C |  | 90 | 7.6 |  | 0.810*** |  |

^a^LAZ, length-for-age Z-score; WAZ, weight-for-age Z-score; AGP, α-1-acid glycoprotein.

^b^C = continuous variable, D = dichotomous variable. ^c^Values are β coefficients, *0.01 ≤ P <0.05, **0.001 ≤ P <0.010, ***P < 0.001, ^a^0.05 ≤ P <0.10. ^d^Measured at study enrolment, ^e^measured at delivery or soon thereafter, ^f^measured at 28 gw, ^g^measured throughout the pregnancy.
